# Life Cycle Assessment of Microplastic Fouling Mitigation Strategies in Membrane Filtration

**DOI:** 10.3390/membranes16040136

**Published:** 2026-04-01

**Authors:** Rendra Hakim Hafyan, Vithushan Indrakumar, Judy Lee, Siddharth Gadkari

**Affiliations:** School of Chemistry and Chemical Engineering, University of Surrey, Guildford GU2 7XH, UK; r.hafyan@surrey.ac.uk (R.H.H.); j.y.lee@surrey.ac.uk (J.L.)

**Keywords:** membrane cleaning, membrane fouling, forward flushing, gas scouring, life cycle assessment

## Abstract

While membrane technologies are critical for preventing microplastics (MPs) release into aquatic ecosystems, MPs-induced fouling remains a persistent bottleneck, necessitating energy-intensive cleaning strategies that introduce their own environmental burdens. This study presents a systematic life cycle assessment (LCA) of fouling mitigation strategies, rigorously comparing hydraulic forward flushing and nitrogen (N_2_) gas scouring across both unmodified and plasma-modified (acrylic acid, cyclopropylamine, and hexamethyldisiloxane) polysulfone membranes. Results reveal a stark divergence between operational performance and environmental sustainability. Baseline operations and the hydraulic flushing of unmodified membranes have environmentally costly global warming potential (GWP) ~150 kg CO_2_-eq/m^3^), driven primarily by high electricity consumption and frequent membrane replacement. Conversely, cyclopropylamine (CPAm) plasma-modified membranes emerging as the optimal strategy, reducing global warming potential to 68 kg CO_2_-eq/m^3^ and cutting electricity demand by 44% through superior fouling resistance. Crucially, the study uncovers a significant trade-off regarding gas scouring: While it achieves the highest technical performance (minimal flux decline of 0.33% h^−1^), the upstream burdens of N_2_ supply increased environmental impacts by over 100% across all categories. These findings challenge the assumption that maximum fouling control equates to sustainability, suggesting that surface engineering via plasma modification, rather than aggressive physical cleaning, offers the most viable pathway for sustainable MPs remediation.

## 1. Introduction

Plastic waste has become a critical environmental concern due to its persistence and associated ecological risks [1]. Wastewater treatment plants (WWTPs) serve as important barriers to plastic emissions, particularly microplastics (MPs) [2]. MPs are defined as plastic particles smaller than 5 mm, which may be intentionally manufactured at this size or generated through the fragmentation of larger plastics (primary and secondary microplastics, respectively) [3]. Their widespread occurrence in the environment is linked to several factors, including their low density and airborne transport potential, which enable dispersion far beyond their original emission sources [4]. MPs are commonly detected in soils through the application of biosolids [5], plastic mulching [6], and atmospheric deposition [7], with their fate further influenced by soil-dwelling organisms and water movement [8]. In aquatic environments, MPs primarily originate from land-based sources, with their transport pathways shaped by hydrodynamic conditions and particle density [9]. Industrial and domestic activities also contribute substantially, as MPs are released via synthetic textile fibers, personal care products, and the degradation of plastic waste [10].

MPs removal in WWTPs occur through primary, secondary, and tertiary processes [11]. Primary treatment, mainly based on sedimentation and flocculation, can remove larger MPs and fibers of 1000 µm, with efficiencies ranging from 65% to 98% [12]. Secondary biological processes, such as activated sludge systems, further reduce MPs by embedding them in microbial flocs, achieving removal efficiencies of approximately 60% to 84% [13]. Tertiary treatment including sand filtration enhance MPs removal and often reach 94% to 98%, effectively polishing effluents before discharge [14]. Although WWTPs are capable of removing a significant proportion of MPs, smaller particles can escape treatment processes and persist in effluents [15]. Among all advanced technologies, membrane-based methods, including MBRs and ultrafiltration, demonstrate the highest removal efficiency by physically retaining particles that would otherwise bypass conventional treatment stages [16]. This underscores the importance of advanced treatment methods, particularly membrane technologies, to enhance MP removal and reduce their release into aquatic systems. Membrane technologies such as microfiltration (MF), ultrafiltration (UF), nanofiltration (NF), and reverse osmosis (RO) are widely applied for MPs remediation, with reported efficiencies in WWTPs reaching up to 99% [2,3,10,17,18].

Despite their effectiveness and wide application for MPs removal in WWTPs, their performance is significantly hindered by fouling [19]. Fouling occurs when various substances accumulate on the membrane surface or penetrate its pore structure, restricting the passage of water [20]. These substances, known as foulants, typically include MPs, organic compounds, and microorganisms [21]. As fouling develops, it leads to pore blockage, cake layer formation, and a reduction in permeate flux, ultimately increasing hydraulic resistance and energy demand during operation [22,23]. Unlike conventional foulants, MPs foulants are highly persistent and non-biodegradable, leading to their continuous accumulation on membrane surfaces [24]. MPs-induced fouling is primarily driven by strong hydrophobic interactions between MPs and membrane surfaces, resulting in firm adhesion [25,26]. Particles smaller than 10 µm can penetrate and block membrane pores, causing irreversible fouling, whereas larger MPs mainly form a porous cake layer through physical adsorption, leading to reversible fouling [27]. Because MPs fouling can be both irreversible and reversible and intensifies over time, regular membrane cleaning is necessary to restore permeability and maintain stable performance [28].

Several cleaning and antifouling strategies have been reported in the literature. Chemical cleaning using alkaline or acidic solutions is widely applied to remove organic and inorganic fouling, but excessive use degrades membrane materials, shortens lifespan, and generates hazardous chemical waste [19,29]. Physical cleaning strategies include mechanical and hydraulic methods, such as backwashing and forward flushing, which use permeate or reverse flow to dislodge cake layers of particle [30,31]. Ultrasonication has emerged as a non-chemical alternative, shown to remove fouling effectively while avoiding secondary pollution associated with conventional chemical cleaning [32]. Gas scouring introduces air or N_2_ bubbles to generate shear forces that dislodge MPs and prevent cake layer formation, though its benefits are less evident for hydrophilic membranes; moreover, evidence on its effectiveness in pressure-driven membranes remains limited [33]. Another strategic approach is to modify the membrane surface to reduce fouling at the material level. Plasma surface modification, for example, enhances membrane hydrophilicity by coating monomers such as acrylic acid (AAc), cyclopropylamine (CPAm), or hexamethyldisiloxane (HMDSO), or through direct plasma treatment, which introduces polar oxygen-containing groups onto the membrane surface. These modifications reduce particle adhesion and improve antifouling performance, providing a proactive complement to conventional cleaning strategies [4,34].

Despite the importance of fouling mitigation in membrane systems, these strategies often require additional energy and material inputs, which in turn impose environmental burden. Life cycle assessment (LCA) is used to evaluate the environmental impacts associated with a product’s life cycle from raw material extraction to recycling/disposal [35]. LCA is increasingly recognized as a valuable tool for evaluating the environmental risk and resource implications of microplastic waste management strategies within a circular economy framework [36]. To date, most environmental impact assessments related to MPs removal have concentrated on WWTPs rather than membrane fouling control. For instance, Puhar et al. [37] conducted an environmental assessment of a pilot plant designed to remove MPs and chemical oxygen demand (COD) from highly contaminated industrial wastewater, achieving removal efficiencies of 98% (mass), 99.9987% (particle count), and 96% COD reduction. LCA revealed that an optimized circular upgrade of the system significantly lowered environmental burdens, including a 96% reduction in global warming potential (GWP) demonstrating its strong sustainability benefits. Jiang et al. [38] developed a green cellulose-membrane strategy for the simultaneous removal of micro- and nano-plastics (MNPs) and heavy metal ions, using bacterial cellulose and attapulgite through vacuum filtration and in situ crosslinking. The membranes achieved >98% MNPs removal and 97% metal adsorption, demonstrated strong tolerance under diverse aquatic conditions, and showed lower environmental impacts than conventional filters according to LCA. Alibekov et al. [3] conducted a LCA of microplastic emissions from WWTPs. The study found that MPs dominated impacts, accounting for 94% of midpoint effects and posing major risks to aquatic species, with polyethylene, polystyrene, and polypropylene as the most harmful polymers. These studies demonstrate the relevance of LCA in evaluating emerging membrane processes for MPs remediation. To the best of our knowledge, no previous studies have systematically quantified and compared the environmental impacts of MPs fouling mitigation strategies in membrane systems.

In this study, we present a life cycle based environmental assessment of MPs fouling mitigation strategies that integrates physical cleaning with plasma engineered membrane surfaces. Forward flushing and N_2_ gas scouring are evaluated as representative hydraulic and two-phase cleaning approaches applied to unmodified and plasma modified polysulfone (PSF) membranes. PSF is selected due to its widespread use in membrane applications, chemical stability, and suitability for plasma surface modification, allowing systematic evaluation of how tailored surface properties influence fouling behavior [39,40,41]. Gas scouring enhances near surface shear through gas liquid flow, disrupting MPs attachment and suppressing cake layer formation, while forward flushing restores permeability by periodically increasing crossflow to remove weakly bound foulants. By explicitly coupling fouling control performance with life cycle impacts, this study moves beyond operational metrics and provides the first quantitative framework for comparing the true sustainability of physical cleaning strategies in membrane-based MPs removal. The results establish how membrane surface chemistry and cleaning intensity jointly govern both fouling behavior and environmental footprint, enabling the evidence-based selection of low impact fouling control pathways for wastewater treatment.

## 2. Materials and Methods

### 2.1. Process Description

**Figure 1:**
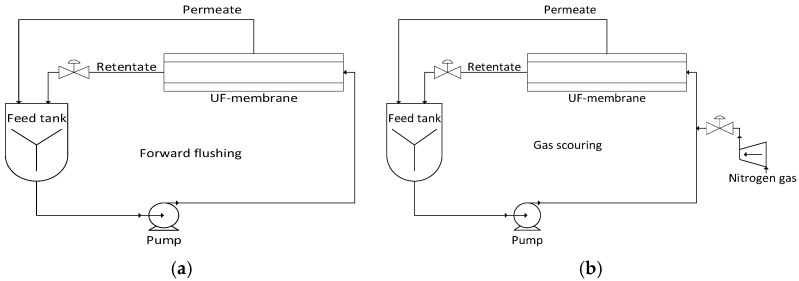
Process flow of NP/MPs cleaning strategies: (**a**) Forwards flushing and (**b**) Gas scouring.

The process description and fouling mitigation information is derived primarily from the work of Enfrin et al. [33] supplemented with insights from related membrane fouling and cleaning studies. The experimental data originate from laboratory-scale investigations and are used here as a conceptual basis for scaling up the membrane cleaning process. The cleaning strategies studied aim to mitigate membrane fouling induced by MPs through periodic physical cleaning approaches, namely forward flushing and gas scouring. These strategies are designed to limit the accumulation of particulate foulants on the membrane surface and to reduce flux decline during operation. Figure 1 depicts a schematic process flow diagram illustrating the two MPs mitigation strategies considered.

Three fouling scenarios are investigated to evaluate membrane performance under different cleaning conditions, as described below.

- No cleaning

In this base scenario, membrane fouling is allowed to develop without the application of any active physical cleaning step. The system is operated under constant filtration conditions. This process serves as a baseline to quantify the extent of fouling and flux decline in the absence of mitigation measures, providing a reference for evaluating the effectiveness of subsequent cleaning strategies.

B. Forward flushing

Forward flushing is a physical cleaning strategy that mitigates MPs-induced fouling by rapidly driving water through membrane in the forward (feed-to-permeate) direction, thereby generating elevated turbulence and hydraulic shear stress at the membrane surface [42]. These conditions promote the detachment of loosely adhered MPs foulants from the membrane surface. The dislodged particles are subsequently transported away from the membrane, reducing the likelihood of the reattachment of pore penetration. As only water is employed during this cleaning step, the operation is classified as a single-phase flow process [33].

C. Gas scouring

Gas scouring is a two-phase physical cleaning strategy in which gas N_2_ bubbles are introduced into the liquid feed stream to enhance shear stress and turbulence at the membrane surface [33]. This approach is particularly effective for mitigating surface fouling caused by MPs, as the rising and sliding gas bubbles generate localized, transient shear forces that disrupt foulant attachment and hinder cake layer formation. In this study, gas scouring is characterized by the gas injection (r), defined as the volumetric fraction of gas relative to the total gas-liquid mixture [43]. Gas injection ratios of r = 0.2 and r = 0.3 are examined, values reported in the literature as effective for enhancing fouling control while maintaining operational stability [33]. Nitrogen is selected as the scouring gas due to its chemical inertness and compatibility with membrane materials [4]. The resulting gas-liquid mixture constitutes a two-phase flow regime, in which bubble-induced turbulence and interfacial interactions weaken the polar and hydrophobic forces binding MPs to the membrane surface [44].

### 2.2. LCA Methodology

This study applies a comparative LCA approach aligned with the ISO14040/14044 framework [45], which defines four phases: Goal and scope definition, life cycle inventory, life cycle impact assessment and interpretation of results.

#### 2.2.1. Goal and Scope Definition

**Figure 2:**
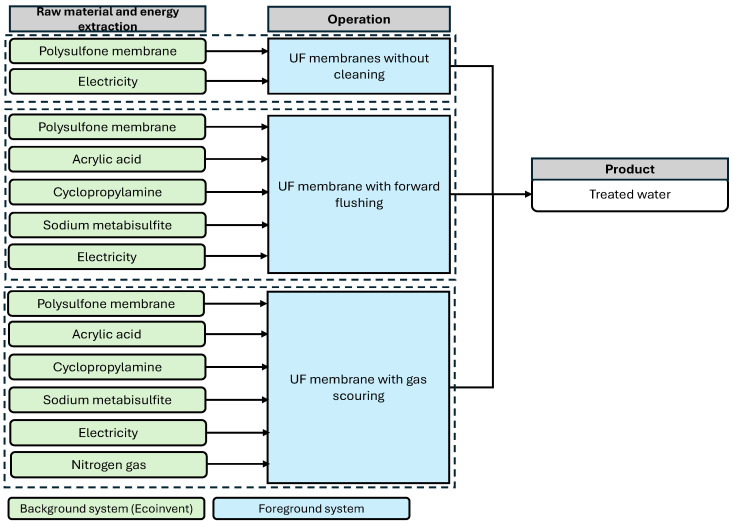
System boundaries of Fouling Mitigation strategies. Note: Surface modification effects are isolated by comparing unmodified and modified membranes within the same physical cleaning boundary (e.g., Scenario 2).

The goal of this study is to evaluate the environmental impacts associated with different membrane fouling mitigation strategies. The assessment focuses on comparing alternative physical cleaning approaches in terms of their environmental performance using the LCA. To ensure practical relevance, the membrane process is scaled to represent a typical small-scale WWTP, with a treatment capacity of approximately 10,000 m^3^, consistent with values reported in the literature [46]. For the purpose of impact assessment, a volume-based functional unit (FU) of 1 m^3^ of treated water, consistent with wastewater LCA literature and providing a standardized reference for cross-scenario comparison [3,47]. A gate-to-gate system boundary is applied, as illustrated in Figure 2, encompassing processes such as raw material consumption and energy supply through to operational activities. Cleaning processes, while capable of partially restoring membrane permeability and flux, incur additional burdens through material and energy consumption. Similarly, membrane replacement introduces further material-related impacts. Downstream end-of-life stages, including chemical waste disposal and membrane recycling or incineration, are excluded. This simplification enhances comparability across scenarios but also represents a limitation, as these processes may contribute significant environmental impacts. A gate-to-gate boundary is selected to support a controlled comparison of fouling mitigation options, focusing on processes that change between scenarios. The study is intended to identify relative trade-offs and hotspots rather than provide a full cradle-to-grave footprint. Processes expected to be broadly similar across scenarios (e.g., infrastructure) or outside the decision control of fouling mitigation selection (e.g., downstream sludge management) are therefore excluded, thereby providing a clearer scientific assessment of the proposed mitigation strategies.

#### 2.2.2. Life Cycle Inventory

Life cycle inventories are compiled using SimaPro datasets to quantify gate-to-gate impacts across all scenarios. Primary data such as membrane materials, compressed N_2_, and chemical use are sourced from peer reviewed literature and directly modelled in SimaPro to ensure transparency and consistency with reported values. Background data including emissions, electricity, and material production are obtained from the Ecoinvent v3.8 database. Table 1 summarizes the compiled and calculated inventory data for the relevant processes. Where data gaps existed, assumptions are applied and explicitly stated to maintain transparency and comparability across scenarios:

- **Operational lifetime:** For comparison purposes, a three-month operational time is adopted as a reference time horizon in the scale-up analysis. This assumption is used solely to standardize the comparison of membrane performance and replacement frequency across different cleaning strategies and does not imply a fixed membrane lifetime.
- **Membrane performance and replacement criteria:**
  1. All membranes are assumed to have an initial permeate flux (J_0_) of 134 L h^−1^ m^−2^, to provide a consistent baseline for evaluating fouling behavior and mitigation performance across different scenarios.
  2. Membrane replacement is assumed to occur when the permeate flux declined to J ≤ 10 L h^−1^ m^−2^.
  3. Each UF module is assumed to have an effective membrane area of 50 m^2^.
- **Membrane production:** Detailed modelling of membrane manufacturing is not undertaken. Instead, the environmental impacts of production are captured through background datasets in SimaPro.
- **Geographical data coverage:** Great Britain (GB) data are used for material and energy inputs where available, while Global (GLO) and Rest of European (RER) data are applied when specific datasets are lacking.
- **Wastewater input:** Influent water is not explicitly modelled, as the study focuses on mitigation strategies and the treated volume remains constant across scenarios.
- **Allocation method:** A cut-off attributional approach is applied, assigning environmental impacts only up to the point materials or processes enter the system. Recycled or reused inputs are treated as burden-free, and no credits are allocated for outputs leaving the system (e.g., recycling or energy recovery) [48].
- **Scenario analysis:** To evaluate the combined effects of membrane surface modification and fouling mitigation strategies, a set of comparative scenarios is developed. The analysis considers both unmodified and plasma-modified PSF membranes operated under different physical cleaning strategies. For gas scouring scenarios, the influence of the gas injection ratio (r) is explicitly examined. The scenarios are organized into four main groups, as outlined below:
  ▪ **Scenario 1 (SC-1):** Baseline (no-cleaning)—no plasma surface modification is applied and a PSF membrane is used. No physical cleaning strategy is implemented, allowing membrane fouling to develop without intervention. This scenario serves as the baseline for evaluating the effects of plasma modification and active fouling mitigation strategies.
  ▪ **Scenario 2 (SC-2):** Fouling control is implemented through periodic forward flushing. The influence of membrane surface properties on fouling mitigation is assessed by comparing the pristine PSF membrane with three plasma-modified variants. Plasma polymerisation is used to tailor surface hydrophilicity and charge, thereby modifying membrane–foulant interactions.
    - Case 2A: PSF membrane—serves as the reference membrane with its original surface chemistry and hydrophobic characteristics, providing a baseline for comparison.
    - Case 2B: PSF + AAc plasma-modified membrane—modified with acrylic acid to increase surface hydrophilicity and introduce negatively charged functional groups, enhancing membrane-water affinity and potentially reducing fouling through electrostatic repulsion.
    - Case 2C: PSF + CPAm plasma-modified membrane—Modified with CPAm to enhance hydrophilicity and introduce positively charged functional groups, allowing the evaluation of electrostatic interactions between the membrane surface and charged foulants.
    - Case 2D: PSF + PSF + HMDSO plasma-modified membrane—Modified with HMDSO to create a hydrophobic layer, enabling the assessment of how increased hydrophobicity influences fouling behavior under periodic flushing.
  ▪ **Scenario 3 (SC-3):** Gas scouring with r = 0.2, indicates a lower gas injection level. The **interaction** between two-phase flow cleaning and membrane surface chemistry is examined across different membrane modifications.
    - Case 3A: PSF membrane
    - Case 3B: PSF + AAc membrane
    - Case 3C: PSF + CPAm membrane
    - Case 3D: PSF + HMDSO membrane
  ▪ **Scenario 4 (SC-4):** Gas scouring with r = 0.3, corresponds to a higher gas injection level. This scenario investigates the effect of a higher gas injection ratio on fouling mitigation performance and environmental impacts.
    - Case 4A: PSF membrane
    - Case 4B: PSF + AAc membrane
    - Case 4C: PSF + CPAm membrane
    - Case 4D: PSF + HMDSO membrane

A fully factorial design would include plasma-modified membranes operated without cleaning to isolate surface modification effects under zero-cleaning conditions. This condition was not available in the underlying experimental dataset used to parameterize flux decline and membrane replacement and was therefore not modelled to avoid introducing unvalidated extrapolations. Attribution of surface modification effects is instead performed via within-regime comparisons (e.g., Case 2A vs. 2B–2D; Case 3A vs. 3B–3D; Case 4A vs. 4B–4D).

#### 2.2.3. Life Cycle Impact Assessment

The environmental impact assessment in this study uses the ReCiPe 2016 method, employing SimaPro v 9.4.0.2 LCA software, which converts life cycle inventory data into impact scores within an internationally recognized and consistent framework. This ensures transparency and comparability across all modelled scenarios. ReCiPe 2016 provides three cultural perspectives: Individualist (I), hierarchist (H), and egalitarian (E) [49]. The individualist perspective reflects short-term interests with a 20-year time horizon but may underestimate long-term impacts. The egalitarian perspective takes a precautionary approach, considering long-term effects over 1000 years and including impact types that are not yet fully established [50]. The hierarchist perspective, which is the most widely applied, balances short- and long-term horizons, drawing on consensus-based scientific models and policy assumptions. A broad set of impact categories is selected to reflect the diversity of processes and materials in the life cycle inventory [51]. These categories include: GWP (kg CO_2_-eq), marine eutrophication (ME, kg N-eq), freshwater ecotoxicity (FE, kg 1,4-DCB), human carcinogenic toxicity (HCT, kg 1,4-DCB), mineral resource scarcity (MRS, kg Cu-eq) and fossil resource scarcity (FRS, kg oil-eq).

## 3. Results and Discussions

### 3.1. Environmental Impact Evaluation

A comparative LCA of the membrane cleaning strategies reveals distinct variations in environmental performance across all evaluated impact categories, as summarized in Table 2. SC-1 represents the baseline condition using an unmodified PSF membrane without any active fouling mitigation. Under this scenario, environmental impacts are primarily driven by severe fouling resulting from the absence of cleaning. The substantial flux decline (76.92 L h^−1^ m^−2^) necessitates frequent membrane replacement and sustained high energy input to maintain operation, thereby increasing the environmental burden. Consequently, this scenario exhibits a GWP of 154.77 kg CO_2_-eq, marine eutrophication of 4.49 × 10^−3^ kg N-eq, freshwater ecotoxicity of 3.16 kg 1,4-DCB, and fossil resource scarcity of 62.83 kg oil-eq. Electricity consumption emerges as a major environmental hotspot in this configuration, largely due to the increased energy demand associated with compensating for flux loss and maintaining system performance [52,53]. This finding aligns with the work of Prézélus et al. [54] reported that approximately 45% of total environmental emissions were attributable to electricity consumption associated with membrane cleaning operations. A marginal improvement is observed in Case 2A, where forward flushing is applied without membrane modification. Environmental impacts decrease slightly (by approximately 1–2% across all categories) compared to SC-1. Although the flux decline remains similar to the baseline, forward flushing intermittently disrupts foulant accumulation, resulting in lower electricity consumption and modest environmental benefits. In contrast, a substantial improvement is achieved in SC-2 with plasma-modified membranes. Surface modification significantly enhances fouling resistance, leading to lower flux decline, reduced membrane replacement frequency, and decreased energy consumption. In particular, Case 2C (PSF + CPAm) and Case 2B (PSF + AAc) exhibit flux decline rates of only 1.33% h^−1^ and 2.33% h^−1^, respectively, as shown in Table 3. These improvements translate into a 40–44% reduction in electricity consumption and a marked decrease in membrane replacement events. By comparison, Case 2D (PSF + HMDSO) shows a more moderate improvement, with a flux decline of approximately 5% h^−1^, resulting in less pronounced environmental benefits. Among all scenarios and cases evaluated, Case 2C demonstrates the lowest overall environmental impact, achieving a GWP of 68.15 kg CO_2_-eq, ME of 1.69 × 10^−3^ kg N-eq, FE of 1.03 kg 1,4-DCB, HCT of 2.00 kg 1,4-DCB, MRS of 0.10 kg Cu-eq, and FRS of 25.33 kg oil-eq. These results highlight the effectiveness of CPAm plasma modification in mitigating fouling, extending membrane lifetime, and reducing energy demand, thereby delivering the most environmentally efficient performance among the scenarios investigated.

A pronounced enhancement in fouling control is observed when transitioning from hydraulic-based strategies (SC-1 and SC-2) to gas scouring–assisted operation in SC-3 and SC-4. The introduction of N_2_ gas scouring substantially reduces flux decline across all cases, with the magnitude of improvement strongly dependent on both the gas injection ratio (r) and membrane surface chemistry. The most effective fouling mitigation is achieved in Case 3C (PSF + CPAm, r = 0.2), Case 4B (PSF + AAc, r = 0.3), and Case 4C (PSF + CPAm, r = 0.3), all of which exhibit an exceptionally low flux decline of 0.33% h^−1^. This represents an order-of-magnitude improvement relative to the baseline (SC-1) and SC-2 cases and directly translates into substantially reduced membrane replacement frequency. From a process-performance perspective, gas scouring combined with plasma-modified membranes therefore provides the most robust fouling control. Despite these operational gains, environmental impacts increase markedly in SC-3 and SC-4. Across all cases, GWP more than doubles relative to SC-1 and SC-2, reaching values between 250.97 and 522.43 kg CO_2_-eq. This increase is not driven by electricity consumption, which is generally lower than in SC-1 and SC-2 due to improved flux stability, but rather by the transportation and consumption of N_2_ gas. This explains why scenarios with the lowest flux decline and membrane replacement frequency nonetheless exhibit the higher environmental impact. Although electricity is a major contributor in membrane systems, intensive N_2_ gas use introduces an additional environmental hotspot under gas-assisted operation. Similarly, Baig et al. [55] identified N_2_ gas and electricity as primary contributors to multiple impact categories in membrane fabrication. Among gas scouring configurations, Case 3C (PSF + CPAm, r = 0.2) demonstrates the best environmental performance, achieving a GWP of 250.97 kg CO_2_-eq, ME of 1.06 × 10^−2^ kg N-eq, FE of 6.39 kg 1,4-DCB, HCT of 10.48 kg 1,4-DCB, MRS of 0.26 kg Cu-eq, and FRS of 58.18 kg oil-eq. These results indicate that moderate N_2_ dosing combined with CPAm modification offers a more balanced trade-off between fouling mitigation efficiency and environmental burden. In contrast, scenarios involving higher gas scouring intensity (SC-4), particularly Case 4A (unmodified PSF), result in the highest environmental impacts across all categories, with a GWP of 522.43 kg CO_2_-eq, ME of 3.08 × 10^−2^ kg N-eq, FE of 18.93 kg 1,4-DCB, HCT of 30.24 kg 1,4-DCB, MRS of 0.73 kg Cu-eq, and FRS of 154.50 kg oil-eq. These elevated impacts arise from the combined effect of high N_2_ supply and less effective fouling resistance, which amplifies N_2_-related emissions without proportionally reducing operational energy or membrane demand. While downstream end-of-life impacts were excluded, it should be noted that the significantly lower replacement frequency of plasma-modified membranes (Cases 2B and 2C) suggests that a full ‘cradle-to-grave’ analysis would likely reveal even greater environmental benefits for surface-engineered systems compared to unmodified baseline operations.

### 3.2. Contribution Analysis

**Figure 3:**
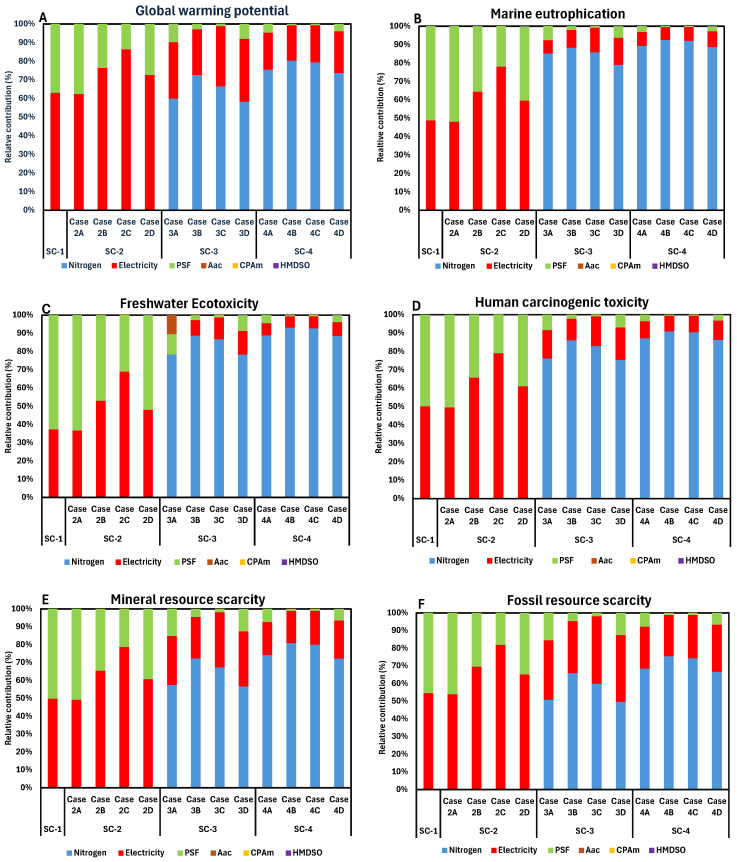
Contribution analysis of different environmental impact categories: (**A**) Global warming potential; (**B**) Marine eutrophication; (**C**) Freshwater ecotoxicity; (**D**) Human carcinogenic toxicity; (**E**) Mineral resource scarcity; and (**F**) Fossil resource scarcity.

A detailed contribution analysis from the LCA of four processes are presented in Figure 3, illustrating how individual unit processes contribute to their overall environmental performance. The key impact categories are selected for further in-depth discussion.

#### 3.2.1. Global Warming Potential

The contribution profile of GWP shifts markedly across scenarios as cleaning strategies intensify. In SC-1 and SC-2, impacts are dominated by electricity (62–76%), followed by PSF membrane use (14–38%), reflecting the energy-intensive operation and frequent membrane replacement under no and forward flushing. Plasma modification chemicals (AAc, CPAm, HMDSO) contribute negligibly (<0.02%), confirming their marginal role in climate impacts. A fundamental transition occurs in SC-3, where N_2_ becomes the dominant contributor, accounting for 58–73% of GWP, while electricity declines to 25–34% and PSF to 1–10%. This dominance intensifies in SC-4, where N_2_ contributes 74–80%, electricity 19–22%, and PSF less than 5%. Despite reduced electricity demand and membrane replacement, the high carbon footprint of N_2_ use and compression outweighs operational savings, explaining the >100% increase in GWP relative to SC-1 and SC-2.

#### 3.2.2. Marine Eutrophication

ME exhibits the strongest sensitivity to N_2_ input among all impact categories. In SC-1 and SC-2, where no N_2_ is supplied, impacts are driven by PSF membrane use (36–52%) and electricity (48–64%), with PSF slightly dominating in several cases. Plasma modification remains insignificant (<0.2%). In SC-3, N_2_ becomes overwhelmingly dominant, contributing 79–88% of total ME, while electricity falls to 10–15% and PSF to 2–8%. This trend is amplified in SC-4, where N_2_ accounts for 89–93% of marine eutrophication across all cases. The higher gas injection ratio directly increases N_2_-related emissions, producing a clear dose-response effect. Notably, AAc- and CPAm-modified membranes exhibit marginally lower PSF and electricity shares, indicating more efficient operation under high N_2_ dosing.

#### 3.2.3. Freshwater Ecotoxicity

In SC-1 and SC-2, freshwater ecotoxicity is primarily associated with PSF membrane use (47–63%), followed by electricity (37–53%). This reflects the toxicity burdens linked to polymer production and upstream material processing rather than energy use alone. With the introduction of gas scouring in SC-3, N_2_ rapidly dominates, contributing 78–89%, while PSF and electricity drop to 3–10% and 9–13%, respectively. In SC-4, N_2_ contribution further increases to 89–93%, confirming N_2_ supply as the primary ecotoxicity hotspot. Plasma modification chemicals again remain negligible (<0.01%), demonstrating that surface functionalization does not introduce additional freshwater toxicity risks.

#### 3.2.4. Human Carcinogenic Toxicity

In SC-1 and SC-2, HCT is shared almost equally between PSF membrane use and electricity consumption, each contributing approximately 50% in the baseline and Case 2A. With plasma-modified membranes in SC-2, electricity becomes dominant, accounting for 61–79%, while PSF contributions decrease to 21–39% due to reduced membrane replacement. Contributions from plasma modification chemicals (AAc, CPAm, HMDSO) remain negligible at <0.02%. A pronounced shift occurs in SC-3, where N_2_ supply dominates, contributing 75–86% of total human carcinogenic toxicity, followed by electricity (12–18%) and PSF (2–8%). This dominance further intensifies in SC-4, with nitrogen contributing 86–91%, electricity 9–11%, and PSF below 4%. The results indicate a strong dependence of carcinogenic toxicity on N_2_ input intensity, while plasma-modified membranes primarily reduce secondary contributions without altering N_2_ dominance.

#### 3.2.5. Mineral Resource Scarcity

MRS shows a more balanced profile in SC-1 and SC-2, with PSF and electricity each contributing approximately 35–51%, depending on membrane type. Plasma-modified cases slightly shift dominance toward electricity (up to 65%), reflecting reduced membrane replacement frequency. In SC-3, N_2_ contributes 57–72% of mineral resource scarcity, surpassing electricity (23–31%) and PSF (1–15%). This pattern persists in SC-4, where N_2_ accounts for 72–81%, driven by mineral-intensive N_2_ production processes. Although membrane replacement is minimized, the upstream resource demand of N_2_ offsets these gains.

#### 3.2.6. Fossil Resource Scarcity

FRS follows a trend similar to GWP but with slightly lower N_2_ dominance. In SC-1 and SC-2, electricity is the leading contributor (54–70%), followed by PSF (30–46%), reflecting fossil fuel use in power generation and polymer synthesis. In SC-3, N_2_ contributes 50–66%, electricity 29–38%, and PSF 1–15%. In SC-4, N_2_ remains dominant at 67–75%, while electricity contributes 23–27%. The results indicate that fossil fuel demand embedded in N_2_ production outweighs savings from reduced electricity consumption and membrane replacement.

## 4. Conclusions

This study provides the first life cycle-based evaluation of microplastic fouling control strategies in membrane filtration, integrating experimental fouling behavior with system level environmental performance. By linking membrane surface chemistry, cleaning intensity, flux decline, membrane lifetime, and upstream material and energy burdens, the work moves beyond laboratory scale performance metrics and establishes how fouling mitigation choices translate into real environmental outcomes. Consistent with prior experimental studies, plasma surface modification, particularly with AAc and CPAm, markedly improves resistance to microplastic fouling by weakening hydrophobic and electrostatic interactions at the membrane surface. These membranes sustain higher flux, require fewer cleaning cycles, and exhibit substantially longer service life than unmodified PSF. When combined with forward flushing, this improved fouling resistance leads to simultaneous reductions in electricity consumption and membrane replacement, yielding the lowest impacts across all assessed categories. This confirms that surface engineered membranes enable a fundamentally more efficient operating regime rather than simply shifting fouling between reversible and irreversible modes. Gas scouring delivers a step change in fouling control, achieving near complete suppression of flux decline and minimal membrane replacement, in line with experimental observations that two-phase flow generates strong near wall shear and disrupts microplastic attachment. However, the life cycle perspective reveals a critical trade off that is not evident from fouling or flux data alone. Nitrogen production and supply dominate every impact category, overwhelming the benefits gained from lower energy use and extended membrane lifetime. As a result, all gas scouring scenarios exhibit substantially higher environmental burdens than hydraulically cleaned, plasma modified membranes, even when fouling control is superior.

These results show that the strategy that maximizes fouling mitigation in the laboratory is not necessarily the one that minimizes environmental impact at scale. The optimal solution emerges not from intensifying physical cleaning, but from combining membrane surface engineering with low energy hydraulic operation. By exposing this disconnect between performance-driven and sustainability-driven optimization, this study provides a quantitative basis for designing next generation membrane systems that control microplastic fouling while remaining environmentally viable.

## Figures and Tables

**Table 1:** Inventory of membrane cleaning strategies for 1 m^3^ of treated water for all investigated scenarios, derived from Enfrin et al. [33].

Input	Unit	SC-1	SC-2				SC-3				SC-4				Database/Sources
			Case 2A	Case 2B	Case 2C	Case 2D	Case 3A	Case 3B	Case 3C	Case 3D	Case 4A	Case 4B	Case 4C	Case 4D	
**Input**															
N_2_	kg	-	-	-	-	-	897.3	816.9	582.6	877.2	1768	1165	1165	1754	Nitrogen, liquid (RER)| market for | Cut-off, S
Electricity	kwh	316.3	308.3	177.2	191	351.4	327.5	200.7	204.6	367.3	338.1	198.9	212	378.4	Electricity, high voltage (GB)| market for | Cut-off, S
PSF	kg	7.3	7.3	2.1	1.2	5.2	4.2	0.9	0.3	3.4	3.1	0.3	0.3	2.6	Polysulfone (GLO)| market for | Cut-off, S
AAc	kg	-	-	1.5 × 10^−3^	-	-	-	5.8 × 10^−4^	-	-	-	1.3 × 10^−4^	-	-	Acrylic acid (RER)| market for acrylic acid | Cut-off, S
CPAm	kg	-	-	-	5.7 × 10^−4^	-	-	-	9 × 10^−5^	-	-	-	9 × 10^−5^	-	Isopropylamine (GLO)| market for | Cut-off, S
HMDSO	kg	-	-	-	-	3 × 10^−3^	-	-	-	2.1 × 10^−3^	-	-	-	1.6 × 10^−3^	Hexamethyldisilazane (GLO)| market for | Cut-off, S
**Output**															
Treated Water	m^3^	1	1	1	1	1	1	1	1	1	1	1	1	1	

**Table 2:** The life cycle impact assessment results per kg of water treated for all the investigated scenarios.

Impact Categories	Units	SC-1	SC-2				SC-3				SC-4			
			Case 2A	Case 2B	Case 2C	Case 2D	Case 3A	Case 3B	Case 3C	Case 3D	Case 4A	Case 4B	Case 4C	Case 4D
Global warming potential	kg CO_2_-eq	154.77	152.32	71.47	68.15	149.28	333.73	250.97	195.21	335.59	522.43	323.23	327.27	521.19
Marine eutrophication	kg N-eq	4.49 × 10^−3^	4.44 × 10^−3^	1.9 × 10^−3^	1.69 × 10^−3^	4.08 × 10^−3^	1.75 × 10^−2^	1.44 × 10^−2^	1.06 × 10^−2^	1.73 × 10^−2^	3.08 × 10^−2^	1.96 × 10^−2^	1.97 × 10^−2^	3.07 × 10^−2^
Freshwater ecotoxicity	kg 1,4-DCB	3.16	3.14	1.24	1.03	2.73	10.90	8.77	6.39	10.65	18.93	11.91	11.96	18.66
Human carcinogenic toxicity	kg 1,4-DCB	5.21	5.14	2.23	2.00	4.76	17.57	14.15	10.48	17.33	30.24	19.11	19.22	29.60
Mineral resource scarcity	kg Cu-eq	0.25	0.25	0.11	0.10	0.23	0.48	0.34	0.26	0.47	0.73	0.44	0.44	0.70
Fossil resource scarcity	kg oil-eq	62.83	61.97	27.61	25.33	58.50	105.54	74.14	58.18	105.69	154.50	92.40	93.82	153.99

**Table 3:** Comparative summary of flux decline across the investigated scenarios.

Scenario		Cleaning Condition	Flux Decline (%/h)
SC-1		No cleaning	6.67%
SC-2	Case 2A	r = 0 (Forward flushing)	6.67%
	Case 2B		2.33%
	Case 2C		1.33%
	Case 2D		5.00%
SC-3	Case 3A	r = 0.2 (Gas scouring)	3.83%
	Case 3B		1.00%
	Case 3C		0.33%
	Case 3D		3.33%
SC-4	Case 4A	r = 0.3 (Gas scouring)	3.17%
	Case 4B		0.33%
	Case 4C		0.33%
	Case 4D		2.67%

## Data Availability

The original contributions presented in this study are included in the article. Further inquiries can be directed to the corresponding author.
